# Mid‐term outcomes of simultaneous coronary artery bypass graft surgery and septal myectomy in patients with hypertrophic obstructive cardiomyopathy: A case‐controlled study

**DOI:** 10.1111/jocs.13988

**Published:** 2019-01-12

**Authors:** Shengwei Wang, Hao Cui, Bing Tang, Changsheng Zhu, Liukun Meng, Qinjun Yu, Xiaohong Huang, Rong Wu, Shuiyun Wang

**Affiliations:** ^1^ Department of Cardiovascular Surgery State Key Laboratory of Cardiovascular Disease Fuwai Hospital, National Center for Cardiovascular Diseases Chinese Academy of Medical Sciences and Peking Union Medical College Beijing China; ^2^ Department of Cardiovascular Surgery Mayo Clinic Rochester Minnesota; ^3^ Department of Special Medical Treatment Center, State Key Laboratory of Cardiovascular Disease, Fuwai Hospital, National Center for Cardiovascular Diseases Chinese Academy of Medical Sciences and Peking Union Medical College Beijing China

**Keywords:** coronary artery disease, coronary artery bypass grafting, hypertrophic cardiomyopathy, septal myectomy

## Abstract

**Background:**

The mid‐term outcome of patients with hypertrophic obstructive cardiomyopathy (HOCM) undergoing coronary artery bypass graft (CABG) is unclear.

**Materials and Methods:**

We studied 44 patients with HOCM and coronary artery disease (CAD) who underwent septal myectomy and CABG) between 2011 and 2017. The control group was matched in a ratio of 4:1 based on age, sex, body mass index, hypertension, and chest pain.

**Results:**

Compared to patients without CAD, patients with CAD had a higher long‐term cardiovascular mortality rate (0.6% vs 6.8%, *P* = 0.03; hazard ratio [HR] = 8.16, 95% confidence interval [CI]: 1.27‐74.48, *P* = 0.03). In addition, 10 out of 176 (5.7%) patients without CAD and nine out of 44 (20.5%) patients with CAD achieved the secondary endpoints (progressive heart failure, unexplained syncope, stroke, atrial fibrillation, and myocardial infarction) (HR = 2.89, 95%CI: 1.03‐8.12, *P* = 0.04). The 5‐year survival rate and cardiovascular event‐free survival rate were significantly higher in patients without CAD than in those with CAD (97.4% vs 93.9%, *P* = 0.03; 89.2% vs 80.1%; *P* = 0.04). In the multivariate analysis, presence of CAD, New York Heart Association class, and left atrial diameter were predictors of combined cardiovascular events when adjusted for age and male sex.

**Conclusions:**

The cardiovascular death and cardiovascular events are significantly increased in patients with HOCM and CAD who underwent CABG at the time of septal myectomy.

## 1 INTRODUCTION

Previous studies have demonstrated that surgical treatment of hypertrophic obstructive cardiomyopathy (HOCM) is associated with a good prognosis.^1^, ^2^ Myocardial ischemia occurring at the microvascular level is a major determinant of the clinical outcome in patients with HOCM, and it is responsible for some of the adverse manifestations of the disease ranging from myocardial infarction to left ventricular (LV) remodeling, systolic dysfunction, ventricular arrhythmias, and even sudden death.^3^ Furthermore, patients with HOCM may also develop coronary atherosclerotic heart disease (CAD). The additional burden of significant obstructive CAD adversely impacts the prognosis of patients with HOCM.^4^ A previous study has reported that although marked symptomatic and hemodynamic benefits can be achieved in patients requiring surgery for HOCM and CAD, the operative mortality rate was higher in patients with CAD because of an increased frequency of iatrogenic ventricular septal defect in these patients.^5^ However, the mid‐term postoperative outcome of patients with HOCM complicated with CAD is unknown. In this study, we sought to evaluate the mid‐term results of patients who have HOCM complicated with CAD.

## 2 METHODS

We retrospective studied 44 patients with HOCM complicated with CAD and 176 patients with isolated HOCM from 867 patients who underwent surgical treatment at Fuwai Hospital in Beijing between 2011 and 2017. This was a single‐center retrospective study. The control group (patients with HCM without CAD, *N* = 176) was generated from the same center and the CAD patients were matched in a ratio of 4:1 based on age, sex, body mass index, hypertension, and chest pain. All patients had undergone coronary arteriography before surgery. The diagnostic criteria and surgical indications of HOCM were consistent with the 2011 American Heart Association/American College of Cardiology guideline and the 2014 European Society of Cardiology guideline, which include unexplained septal hypertrophy with a thickness >15 mm.^6^, ^7^ The indications for septal myectomy were (1) severe symptoms or syncope or near‐syncope despite optimal medical therapy and (2) a LV outflow tract (LVOT) gradient >50 mm Hg at rest or with provocation. All coronary stenoses >50% were bypassed. Patients with myocardial bridging were excluded.

All patients signed informed consent forms, including those for their biomarker analysis and clinical data, prior to enrollment. The study was approved by The Ethics Committee of Fuwai Hospital. All procedures were conducted in accordance with the ethical principles stated in the Declaration of Helsinki.

## 3 SURGICAL TECHNIQUE

A standard median sternotomy was performed in all patients. Cardiopulmonary bypass was performed using ascending aortic cannulation and bicaval cannulation. Myocardial protection was achieved with antegrade, cold blood cardioplegia. The aorta was transected approximately 7 mm above the right coronary ostium. The left‐right sinus and right non‐sinus commissures were elevated and retracted with 5‐0 Prolene suture. Auxiliary light and loupe magnification were used to achieve better inspection of the left ventricular cavity. The resection ranges were as follows: The upper end was located at approximately 4 mm below the aortic ring; the lower end was extended to the apex of the left ventricle; from the right side, the myectomy was started slightly rightward to the nadir of the right aortic cusp; and to the left, resection was terminated near the mitral anterior commissure. Elongated leaflets were not routinely folded, unless severe regurgitation or systolic anterior motion (SAM) remained after resection. The detailed procedure is described in our previous publication.^8^ The coronary artery bypass graft (CABG) procedure was performed.

## 4 STUDY ENDPOINTS

The primary clinical endpoints were cardiovascular mortality, including: 1) sudden cardiac death (SCD; unexpected, within 1 h of a witnessed collapse or nocturnal death in previously stable patients); 2) heart failure‐related death occurring in patients who had progressive cardiac decompensation ≥1 year before death; 3) stroke‐related death due to a probable or proven embolic stroke; 4) aborted cardiac arrest or appropriate discharge of an implantable cardioverter defibrillator for ventricular fibrillation; and 5) heart transplantation for drug‐refractory heart failure (these deaths were recorded as surrogate heart failure‐related deaths). The secondary endpoints were cardiovascular‐related morbidity involving unexplained syncope, non‐sustained ventricular tachycardia, atrial fibrillation, mural thrombus, embolic stroke, transient ischemic attack, myocardial infarction, or progressive heart failure with an increase of at least one New York Heart Association (NYHA) functional class and readmission owing to cardiovascular complications. Follow‐up was performed at the time of admission and was performed via subsequent clinic visits and telephone calls. Clinical and echocardiographic data were collected during the latest clinic visits.

## 5 STATISTICAL ANALYSES

The results are expressed as mean ± standard deviation, median (interquartile range), or percentage, as appropriate. Student's *t*‐test for independent samples and the Mann–Whitney *U* test were used to compare continuous variables, and the χ^2^ or Fisher exact test was used to compare nominal variables, as appropriate. Propensity score matching (PSM) were used to balance various risk factors of CAD. The propensity score was calculated using a logistic regression model with 1:4 nearest neighbor matching without replacement based on a caliper width of 0.2. The Kaplan‐Meier method was used to calculate survival free from the endpoint events. A log‐rank test was used to compare the survival curves between patients with and without CAD. Differences in baseline characteristics between the groups were adjusted using multivariate Cox models. A stepwise multiple Cox analysis technique were used to identify the variables independently associated with the endpoints in these analyses that were incorporated into the final models. Variables with *P* < 0.1 on univariate analysis were entered into a multivariate analysis. All reported probability values were 2‐tailed, and a *P*‐value <0.05 was considered statistically significant. SPSS version 24.0 statistical software (IBM Corp., Armonk, NY) and Prism GraphPad 7.0 (GraphPad Software Inc., La Jolla, CA) were used for calculations and illustrations, respectively.

## 6 RESULTS

We enrolled 220 patients who underwent septal myectomy or septal myectomy and CABG. The baseline characteristics of the patients according to the absence or presence of CAD are described in Table 1. The mean age of the patients was 55.4 ± 9.2 years. Compared to patients without CAD, the levels of brain natriuretic peptide (BNP; 1922.8 ± 1894.5 vs 1072.2 ± 1010.8, *P* = 0.02) and the left ventricular end‐diastolic diameter were significantly higher in patients with CAD (42.4 ± 3.6 vs 44.7 ± 4.7, *P* = 0.01). There was no difference in the other parameters between the groups; 47.8% patients had three or four diseased vessels and only 13.6% patients had one diseased vessel.

**Table 1:** Baseline patient characters

Variable	Without CAD (*n* = 176)	With CAD (*n* = 44)	*P‐*value
Age, (years)	55.3 ± 9.4	55.7 ± 8.0	0.801
Male, *n*	138 (78.4%)	33 (75.0%)	0.870
Body mass index, kg/m^2^	26.8 ± 3.3	27.3 ± 4.2	0.513
Family history of HCM or SCD, *n*	26 (14.8%)	10 (22.7%)	0.202
Heart rate, beats/min	72.4 ± 9.7	72.9 ± 9.7	0.742
NYHA class	2.9 ± 0.5	2.9 ± 0.4	0.577
BNP, pg/mL	1072.2 ± 1010.8	1922.8 ± 1894.5	0.02
History of smoking	26 (14.8%)	7 (15.9%)	0.816
Comorbidities			
Hypertension, *n*	76 (43.2%)	21 (47.7%)	0.587
Hyperlipidemia, *n*	25 (17.6%)	6 (13.6%)	0.528
Diabetes mellitus, *n*	10 (6.8%)	4 (9.1%)	0.604
Clinical presentation			
Chest pain, *n*	56 (31.8%)	16 (36.4%)	0.771
Amaurosis, *n*	20 (11.4%)	5 (11.4%)	1.00
Syncope, *n*	24 (13.6%)	7 (15.9%)	0.698
Chest distress, *n*	103 (58.5%)	27 (61.4%)	0.732
Echocardiographic indices			
Aorta, mm	32.7 ± 4.4	31.9 ± 4.4	0.636
Left atrium, mm	46.4 ± 7.7	44.0 ± 7.0	0.120
LVEDD, mm	42.4 ± 3.6	44.7 ± 4.7	0.01
IVST, mm	22.3 ± 4.3	23.3 ± 5.5	0.127
Posterior LV wall, mm	12.3 ± 2.3	12.1 ± 2.2	0.823
LVOTG, mmHg	85.5 ± 26.8	77.7 ± 26.2	0.087
LVEF, %	70.9 ± 6.0	72.3 ± 6.2	0.125
Mitral regurgitation*	1.9 ± 0.7	1.7 ± 0.9	0.153
Medical therapy			
Beta‐blockers, *n*	128 (72.7%)	30 (68.2%)	0.549
Calcium‐channel blockers, *n*	22 (12.5%)	2 (4.5%)	0.178

Values are presented as percentage, mean ± standard deviation, or median (interquartile range) when appropriate.

BNP, brain natriuretic peptide; CAD, coronary artery disease; HCM, hypertrophic myocardiopathy; IVST, interventricular septal thickness; LV, left ventricular; LVEF, left ventricular ejection fraction; LVEDD, left ventricular end‐diastole diameter; LVOTG, left ventricular outflow tract gradient; NYHA, New York Heart Association; SCD, sudden cardiac death.

* Scoring of mitral regurgitation: 0 = none, 1 = mild, 2 = moderate, 3 = severe.

Compared to patients without CAD, the cardiopulmonary bypass time (131.4 ± 65.8 vs 108.5 ± 48.4, *P* = 0.03) and aortic cross‐clamping time (87.9 ± 39.9 vs 72.8 ± 37.7, *P* = 0.02) were significantly higher in patients with CAD. No perioperative death was observed in any of the groups. No difference was found between the groups in regard to hospital stay, postoperative hospital stays, ventilation time, and concomitant operations (Table 2). Number of vessels bypassed were 2.4 ± 1.0 and the use of the internal mammary artery is described in Table 3. Only eight (18.2%) patients had one coronary artery bypass graft, and 17 (38.6%) patients had three coronary artery bypass grafts. The number and location of diseased vessels are shown in Table 3.

**Table 2:** Perioperative data

Variable	Without CAD (*n* = 176)	With CAD (*n* = 44)	*P*‐value
Concomitant procedures			
Mitral valvuloplasty, *n*	26 (14.8%)	5 (11.4%)	0.561
Mitral valve replacement, *n*	4 (2.3%)	1 (2.3%)	1.00
Double valve replacement, *n*	1 (0.6%)	1 (2.3%)	0.361
Tricuspid valvuloplasty, *n*	16 (9.1%)	3 (6.8%)	0.77
WPW pathway amputation, *n*	1 (0.6%)	0 (0%)	1.00
Cox Maze procedure, *n*	17 (9.7%)	2 (4.5%)	0.280
Cardiopulmonary bypass time, min	108.5 ± 48.4	131.4 ± 65.8	0.03
Aortic crossclamping time, min	72.8 ± 37.7	87.9 ± 39.9	0.02
Postoperative ventilation time, min	24.3 ± 33.0	24.2 ± 21.4	0.99
Postoperative hospital stays, day	9.1 ± 5.7	10.1 ± 9.5	0.37
Pacemaker implantation	3 (1.7%)	1 (2.3%)	0.80

Values are presented as percentage, mean ± standard deviation, or median (interquartile range) when appropriate. CAD, coronary artery disease; WPW, Wolff‐Parkinson‐White.

**Table 3:** Coronary artery bypass procedures and conduits

Procedure	Number of patients	Conduits of CABG	Number of patients
CABG × 1, %	8 (18.2%)	LIMA, %	26 (63.6%)
CABG × 2, %	14 (31.8%)	BIMA, %	0 (0%)
CABG × 3, %	17 (38.6%)	RA, %	0 (0%)
CABG × 4, %	4 (9.1%)	SVG, %	34 (77.3%)
CABG × 5, %	1 (2.3%)	Single arterial grafts, %	26 (63.6%)
		Multiple arterial grafts, %	0 (0%)

BIMA, bilateral internal mammary artery; CABG, coronary artery bypass; LIMA, left internal mammary artery; RA, radial artery; SVG, saphenous vein graft.

**Figure 1:**
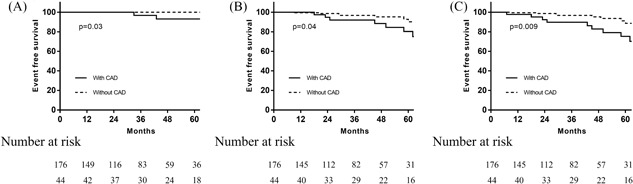
Kaplan‐Meier analysis of primary, secondary, and combined end points between the two groups. A, Primary end points between CAD and without CAD group; (B) Secondary end points between CAD and without CAD group; (C) combined end points between CAD and without CAD group. CAD, coronary artery disease

As shown in Table 4, the mean follow‐up duration (37.0 [interquartile range (IQR) 23.0‐57.0] vs 48.5 [IQR, 25.7‐64.0], *P* = 0.07) was similar in the two groups. During the follow‐up, a total of four patients achieved the primary endpoints. Heart failure‐related death occurred in one patient without CAD and in one patient with CAD, and sudden cardiac death occurred in two patients with CAD (Table 4). Compared to patients without CAD, those with CAD had a higher cardiovascular mortality rate (0.6% vs 6.8%, *P* = 0.03). The incidence of cardiovascular‐related death was worse in patients with CAD (hazard ratio [HR] = 8.16, 95% confidence interval [CI]: 1.27‐74.48, *P* = 0.03). The 5‐year survival rate was significantly higher in patients without CAD than in those with CAD (97.4% vs 93.9%, *P* = 0.03) (Figure 1A). In addition, the LVOT gradient, NYHA class, degree of mitral regurgitation, and LVOT were significantly improved after the surgery. There was no difference about the incidence of postoperative LVOT gradient >30 mmHg and systolic anterior motion between the two groups (Table 5).

**Table 4:** Follow‐up data

Variable	Without CAD (*n* = 176)	With CAD (*n* = 44)	*P*‐value
Follow‐up months	37.0 (23.0–57.0)	48.5 (25.7–64.0)	0.07
Primary end points	1 (0.6%)	3 (6.8%)	0.03
SCD, *n*	0 (0%)	2 (4.5%)	0.04
HF‐related death, *n*	1 (0.6%)	1 (2.3%)	0.36
Secondary end points	10 (5.7%)	9 (20.5%)	0.002
Progressive HF, *n*	6 (3.4%)	2 (4.5%)	0.66
Unexplained syncope, *n*	0 (0%)	1(0.6%)	0.20
Stroke, *n*	2 (1.1%)	2 (4.5%)	0.38
Atrial fibrillation, *n*	2 (1.1%)	3 (6.8%)	0.05
Myocardial infarction, *n*	0 (0%)	1 (0.6%)	0.20

Values are presented as percentage. CAD, coronary artery disease; HF, heart failure; SCD, sudden cardiac death.

**Table 5:** Clinical and echocardiographic data at last follow‐up visit

Variables	Without CAD (*n* = 176)	With CAD (*n* = 44)	*P*‐value
NYHA class	1.1 ± 0.4	1.2 ± 0.5	0.261
Max LVOT gradient >30 mmHg	10 (5.7%)	2 (4.5%)	0.762
Mitral regurgitation*	0.77 ± 0.79	0.75 ± 0.81	0.901
Max IVST, mm	14.2 ± 3.4	14.5 ± 3.2	0.640
LVEF, %	64.8 ± 5.4	64.4 ± 5.3	0.718

*Scoring of mitral regurgitation: 0 = none, 1 = mild, 2 = moderate, 3 = severe. CAD, coronary artery disease; MB, myocardial bridging; NYHA, New York Heart Association; IVST, interventricular septal thickness; LVEF, left ventricular ejection fraction; LVOT, left ventricular outflow tract gradient; LVWT, left ventricular wall thickness.

A total of 19 patients achieved the secondary endpoints, including 10 out of 176 (5.7%) patients without CAD and nine out of 44 (20.5%) patients with CAD (HR = 2.89, 95%CI: 1.03‐8.12, *P* = 0.04) (Figure 1B). Kaplan‐Meier analysis demonstrated that the 5‐year cardiovascular event‐free survival rate of patients without CAD was higher than that of patients with CAD (89.2% vs 80.1%; *P* = 0.04) (Figure 1B). The 5‐year cardiovascular event‐free survival rate was significantly higher in patients without CAD than in patients with CAD (89.2% vs 80.1%, *P* = 0.009) (Figure 1C). In the multivariate analyses, the presence of CAD was predictive of combined cardiovascular events adjusted for age, male sex, and body mass index. Additional significant covariates for the combined endpoint were higher NYHA class and left atrial diameter (Table 6).

**Table 6:** Multivariate Cox proportional hazards models for the all cause death

	Univariable		Multivariate	
Variable	RR (95%CI)	*P*	RR (95%CI)	*P*
Age	0.99 (0.89–1.10)	0.915		
Male	0.89 (0.10–7.96)	0.916		
Body mass index	1.20 (0.97–1.48)	0.09		
NYHA class	1.26 (1.04–1.51)	0.02	1.49 (1.12–1.99)	0.006
Left atrial diameter	1.13 (1.009–1.27)	0.04	1.18 (1.02–1.36)	0.02
Mitral regurgitation	1.31 (1.07–1.46)	0.09		
Postoperative hospital stays	1.04 (1.00–1.08)	0.08		
Interventricular septal thickness	1.14 (0.99–1.33)	0.08		
Concomitant CABG	1.66 (1.07–2.58)	0.02	1.76 (1.11–2.81)	0.02

CABG, coronary artery bypass graft; CI, confidence interval; NYHA, New York Heart Association; RR, relative risk.

## 7 DISCUSSION

Patients with HOCM may also develop CAD, and up to 20% prevalence of CAD in these patients has been reported.^9^, ^10^ In the present study, patients with HOCM and CAD were generally older than those with isolated HCM, which is consistent with a previous study.^1^ Furthermore, we found that compared to patients without CAD, those with HOCM and CAD had a higher BNP level. This could be attributed to the presence of CAD and has further contributed to the diastolic dysfunction.^11^ In addition, we found that in patients with CAD, the left ventricular end‐diastolic diameter was significantly greater than that in patients without CAD, which is a marker for disease severity in HCM.^12^ These results were consistent with those of a previous study that reported that the additional burden of severe CAD adversely contributed to the development of HOCM.^13^

Myocardial ischemia frequently occurs in patients with HCM. The main mechanisms underlying its development include decreased coronary flow reserve, disease of small intramuscular arteries, “inadequate” size of coronary arteries relative to the hypertrophied myocardium, diminution of coronary flow and compression of septal perforator arteries during systole, and co‐existent atherosclerotic CAD, which can be present in up to a quarter of patients with HCM who are older than 45 years.^14^ The presence of myocardial ischemia plays an important role in the pathophysiology of HCM.^15^ Previous studies have found that the presence of myocardial bridging in adult patients with HCM does not appear to increase the risk of overall mortality and SCD.^16^, ^17^ However, in contrast, a previous study has found that the additional presence of CAD does have a marked detrimental impact on prognosis, likely via an interaction with the pathophysiological abnormalities of HCM as opposed to the independent effects of coronary atherosclerosis or HCM.^4^ In the present study, none of the patients who underwent CABG and septal myectomy or isolated septal myectomy during the perioperative period experienced severe complications, which is inconsistent with a previous study that reported that concomitant CABG was associated with a higher prevalence of iatrogenic ventricular septal defect. In addition, this is inconsistent with the previous study that the risk of hospital death after isolated septal myectomy for obstructive HCM is <1%. This inconsistency could be attributed to the technological advances in surgery over the last few years and the relatively small number of patients who were matched with HOCM and CAD.^18^ However, we found that a significantly higher number of postoperative mid‐term cardiovascular‐related deaths and adverse cardiovascular events was observed in patients with HOCM complicated with CAD than in those without CAD. Patients with combined procedures such as mitral surgery, Maze, or aortic valve surgery may contribute to an adverse result in previously reported HOCM patients.^2^ No difference in the incidence of concomitant surgery between the two groups and multivariable Cox analysis revealed that only CAD, higher NYHA class, and left atrial diameter were associated with poorer outcome in HOCM patients. These results indicate that the presence of CAD can significantly reduce the mid‐term survival of these patients even after CABG and septal myectomy.

The present study has some limitations. First, the study was conducted retrospectively in a single tertiary center, which might have led to a selection bias. Second, number of patients with HOCM complicated with CAD enrolled in this study was limited. We did not compare the mid‐term outcomes of patients with isolated CAD with the outcomes of patients with HOCM and CAD in our center.
